# Commissioning and performance evaluation of commercially available mobile imager for image guided total body irradiation

**DOI:** 10.1002/acm2.13865

**Published:** 2022-12-26

**Authors:** Tetsu Nakaichi, Hiroyuki Okamoto, Mitsuhiro Kon, Kazuki Takaso, Ako Aikawa, Satoshi Nakamura, Kotaro Ijima, Takahito Chiba, Hiroki Nakayama, Mihiro Takemori, Shohei Mikasa, Kyohei Fujii, Yuka Urago, Tomonori Goka, Yuri Shimizu, Hiroshi Igaki

**Affiliations:** ^1^ Radiation Safety and Quality Assurance Division National Cancer Center Hospital Chuo‐ku Tokyo Japan; ^2^ Department of Radiological Technology Radiological Oncology National Cancer Center Hospital Chuo‐ku Tokyo Japan; ^3^ Department of Radiological Sciences Graduate School of Human Health Science Tokyo Metropolitan University Arakawa‐ku Tokyo Japan; ^4^ Department of Radiation Sciences Komazawa University Setagaya‐ku Tokyo Japan; ^5^ Department of Radiation Oncology National Cancer Center Hospital Chuo‐ku Tokyo Japan

**Keywords:** EPID, in vivo dosimetry, lung shield, mobile imager, real‐time monitoring, TBI

## Abstract

**Background:**

The setup of lung shield (LS) in total body irradiation (TBI) with the computed radiography (CR) system is a time‐consuming task and has not been quantitatively evaluated. The TBI mobile imager (TBI‐MI) can solve this problem through real‐time monitoring. Therefore, this study aimed to perform commissioning and performance evaluation of TBI‐MI to promote its use in clinical practice.

**Methods:**

The source‐axis distance in TBI treatment, TBI‐MI (CNERGY TBI, Cablon Medical B.V.), and the LS position were set to 400, 450, and 358 cm, respectively. The evaluation items were as follows: accuracy of image scaling and measured displacement error of LS, image quality (linearity, signal‐to‐noise ratio, and modulation transfer function) using an EPID QC phantom, optimal thresholding to detect intra‐fractional motion in the alert function, and the scatter radiation dose from TBI‐MI.

**Results:**

The accuracy of image scaling and the difference in measured displacement of the LS was <4 mm in any displacements and directions. The image quality of TBI imager was slightly inferior to the CR image but was visually acceptable in clinical practice. The signal‐to‐noise ratio was improved at high dose rate. The optimal thresholding value to detect a 10‐mm body displacement was determined to be approximately 5.0%. The maximum fraction of scattering radiation to irradiated dose was 1.7% at patient surface.

**Conclusion:**

MI‐TBI can quantitatively evaluate LS displacement with acceptable image quality. Furthermore, real‐time monitoring with alert function to detect intrafraction patient displacement can contribute to safe TBI treatment.

## 1 INTRODUCTION

Total body irradiation (TBI) was administrated as conditioning regimens before a hematopoietic stem cell transplantation for various hematological diseases, including leukemia, lymphoma, myelodysplastic syndrome, and severe aplastic anemia.^1^, ^2^, ^3^ A total of 12 grays (Gy) in six fractions was the most frequently used myeloablative TBI to eliminate malignant cells.^2^, ^3^, ^4^ In reduced‐intensity stem cell transplantation, a low dose regimen of 2–4 Gy is mainly recommended for elderly patients and those with poor general condition.^5^

To uniformly irradiate the entire body of the patient, various techniques such as long source‐to‐surface distance (LSSD), translational couch,^6^, ^7^ helical tomotherapy,^8^, ^9^ and volumetric intensity modulated arc therapy ^10^, ^11^ have been developed.

Among these, LSSD TBI is a simple technique that can be used in many facilities and has been used in approximately 90% of facilities performing TBI.^1^ Compensation for differences in absorbed thickness and reduction of lung dose in the LSSD TBI technique are achieved using an absorber such as compensators,^12^, ^13^, ^14^ lung shield (LS),^3^, ^4^, ^5^, ^6^, ^7^, ^8^, ^9^, ^10^, ^11^, ^12^, ^13^, ^14^, ^15^ field in field (FIF),^16^, ^17^ and intensity‐modulated radiation therapy (IMRT) ^18^ to consider organ geometry. Computed radiography (CR) images have been mainly used to ensure the position of the compensator; however, identifying the amount of movement is a time‐consuming task.^19^ Furthermore, it would not be suitable for FIF and IMRT, which require multiple treatment field checks.

In recent years, dedicated TBI imagers have been developed to confirm the position of the shield, treatment field, and monitoring during prolonged TBI treatment.^16^, ^17^, ^18^, ^19^ Dipasquale et al. developed an electronic portal imaging device (EPID) composed of amorphous silicon that automatically moves along the rail.^19^ They reported that the image acquisition time was reduced by up to 90%, and the LS positioning was more significantly improved than a CR system. Other studies using a commercially available mobile imager have shown their clinical usefulness in image‐guided TBI including FIF ^16^, ^17^ and a step and shoot IMRT ^18^ to ensure multi‐leaf collimator (MLC) field, collimator angle, and patient position based on the treatment field.

However, to our knowledge, the commercial TBI mobile imager (CNERGY TBI, Cablon Medical B.V.) for quantitative setup with real‐time monitoring has not yet been fully commissioned or physically evaluated.

Thus, this study aimed to perform commissioning of commercially mobile imager for TBI by evaluating its physical characteristics, including the accuracy of detecting intra‐fractional errors, image quality, determination of the optimal threshold for alerting functions, and scattered radiation from the imager.

## 2 MATERIALS AND METHODS

### 2.1 Linear accelerator

Data were acquired using Clinac iX (Varian Medical Systems, Palo Alto, California, USA) equipped with the Millennium 120 leaf MLC. The beam energy and dose rates of photon beam available at our hospital were 100, 150, 200, and 250 monitor unit (MU)/min in 4 MV and 100, 200, 300, 400, 500, and 600 MU/min in 10 MV.

### 2.2 TBI treatment

The beam energy and dose rate for TBI treatment are 10 MV and 300 MU/min (SAD 100 cm), respectively, accounting for clinical efficiency and radiation‐induced toxicities. The field size was 40 × 20 cm in horizonal and vertical directions, respectively, at SAD 100 cm, and the collimator angle was 0°. During TBI treatment, the patient is in the supine position with arms crossed over the abdomen and knees lightly bent. A day before the first day of TBI treatment, the patient is fixed with a thermoreversible shell (Polyform, SAKAI Medical Co., Ltd., Tokyo, Japan) to position the LS in the lung. With the LS in place, two or three CR images are considered to position the LS and the shell is marked with reference to the light field. To reduce the lung dose, the LS is used on the first and final day of 12 Gy in six fractions (two fractions per day). The LS shape is made by contouring the lungs on the digitally reconstructed radiograph image reconstructed from the CT image and imported into the treatment planning system (Eclipse version 15.6, Varian Medical Systems, Palo Alto, California, USA). The LS thickness is made using a 1.8 cm Cerrobend, which is determined by measuring the half‐layer of a 10‐MV photon beam. The mean lung dose estimated using the LS is approximately 8 Gy in 12 Gy per six fractions.

### 2.3 Mobile imager

The CNERGY TBI is a stand‐alone mobile imager with a large image area that allows real‐time monitoring during TBI treatment. The detector consists of a charge coupled device (CCD) camera with a pixel size of 6.7 × 6.7 μm and a resolution of 1280 × 1024. The imaging area was 75 × 46 cm in horizonal and vertical directions, respectively. Images are acquired every second during treatment and can be stored at any second to verify patient and LS positions. The difference between the patient's current and reference positions is always displayed in a colored image. The final image determining the LS position is used as a reference position image. An audible alarm sounds when the threshold for the difference in images exceeds the predefined threshold. The workflow efficiency in identifying the LS position and quantitatively assessing the treatment setup accuracy has been reportedly improved, and the intra‐fractional motion through real‐time monitoring can be minimized during treatment.

### 2.4 Comparison the required time using CR to TBI imager

To compare the total required time for imaging procedure with CR and TBI imager when using LS (first day and final day in 12 Gy per six fractions), we investigated the required time for each procedure and calculated the total required time in each fraction (1, 2, and 3) with considering the repeated procedures.

### 2.5 Evaluation of the mobile imager characteristics

Physical evaluations were performed at source‐axis distance (SAD) of 400 cm with patient axis position, SAD of 358 cm with the LS, and SAD of 450 cm with CNERGY TBI. Measurements were made using a distance meter with an infrared sensor (Bosh, Zamo, Robert Bosch Tools Gmbh, 70538 Stuttgart, Germany).

### 2.6 Accuracy of image scaling

The vertical and horizonal lengths of the square phantom were measured to determine the scaling ratio using a caliper. Then, it was placed at the LS position (SAD, 358 cm). The scaling phantom was irradiated for 10 s using a 10‐MV photon beam with a dose rate of 300 MU/min, and approximately 10 images were acquired. An image was selected from ten images by visually evaluating the image quality. The number of vertical and horizontal pixels in the selected image were measured five times. The scaling ratio (cm/pixels) was calculated from the measured length using the caliper and average number of pixels. Results of the scaling ratio (0.437 cm/pixels in vertical and horizonal directions) were registered to a mobile imager system to obtain absolute position on images.

Then, to confirm the accuracy of the measured length at the calibration position, the scaling phantom was again irradiated at the same position (SAD, 358 cm) and was measured five times. Furthermore, measurements were taken at four different positions ± 1 cm, ± 2 cm from the calibration position because the LS placement slightly differs along the beam axis in clinical practice. The vertical and horizontal lengths of the scaling phantom were measured five times from images at each location, and differences from the reference length (22.8 × 23.2 cm) measured using the calipers were calculated.

### 2.7 Accuracy of measured lung shield displacement

**FIGURE 1:**
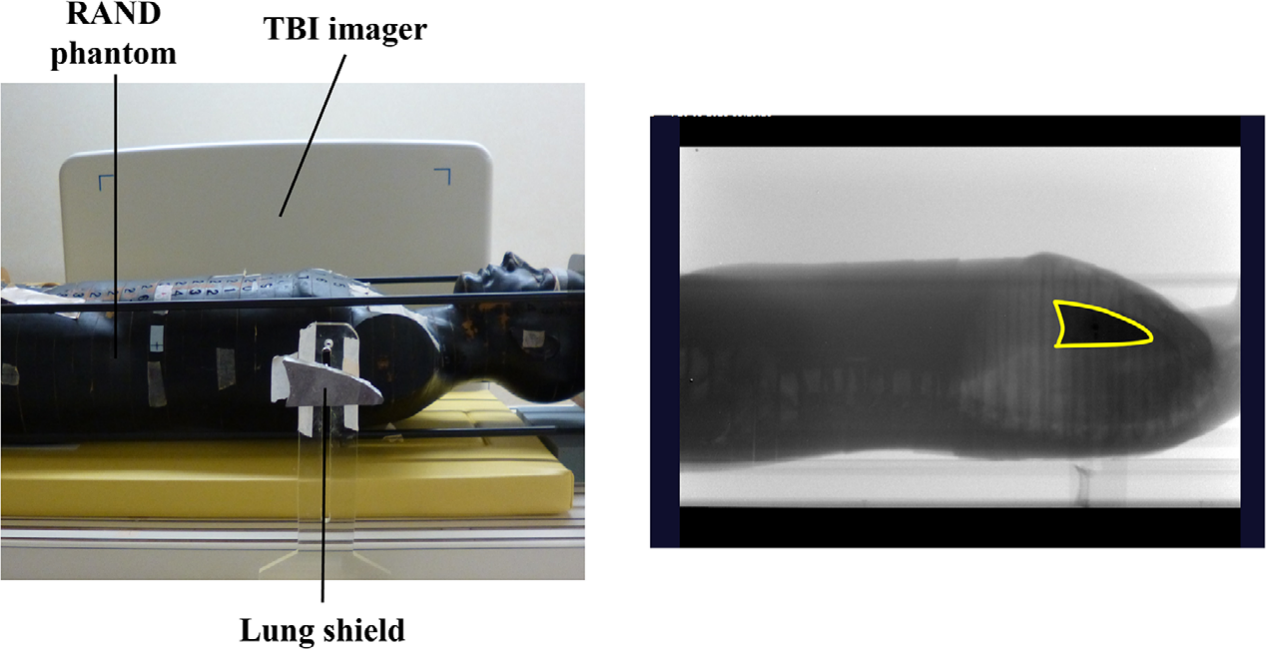
Left: a setup geometry of an anthropomorphic RANDO phantom and the LS placed on the dedicated TBI couch; right: an image of RANDO phantom with the LS placed taken by TBI imager and manually outlined the LS (yellow line). RANDO; LS; lung shield

**FIGURE 2:**
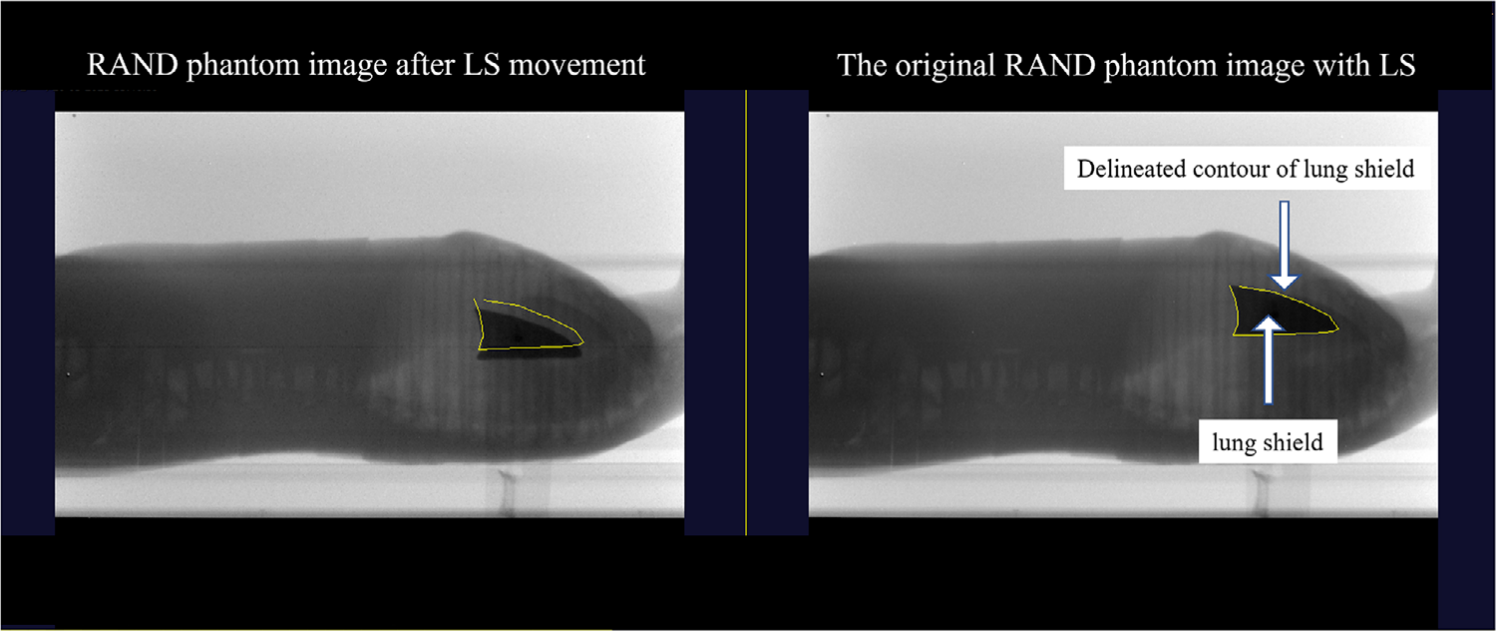
Images with RANDO phantom using a lung shield obtained by TBI imager. Left: example of an image with the LS displaced 1‐cm posterior from the reference position; right: example of RANDO phantom image acquired with the LS placed at the reference position and manual contouring of the LS

Figure 1 shows an anthropomorphic RANDO phantom (SAD of 400 cm, The Phantom Laboratory, Ltd., New York, USA) and the LS (SAD of 358 cm) placed on the dedicated TBI couch (the Model ORP‐TBI‐MN, Orion Electric Co., Ltd., Nagoya, Japan) and acquired image using the systems. The phantom and LS placements were arranged corresponding to image center using the light field, and a 10‐MV photon beam with dose rate of 300 MU/min was irradiated for 10 s. An image was selected as reference to delineate the LS contour (Figure 2). Then, the LS was intentionally moved by 5, 10, and 20 mm in anterior–posterior and superior–inferior directions, respectively, and images were obtained five times. The contour of the LS location can be manually adjusted to match the image contour obtained after the LS movement, and the displacement due to matching is displayed. Regarding displacements in each direction, the difference between the known and measured displacements was measured five times. The mean and standard deviation of the difference error were calculated.

### 2.8 Image quality of TBI imager

The quality of the stored image (frame rate: 1 image/s), was measured using EPID QC phantom (PTW‐Freiburg GmbH, Freiburg, Germany), placed at SAD of 400 cm. Therefore, the image quality depends on different dose rates, and better image quality is expected in higher dose rates. To compare the image quality at different dose levels, the phantom was irradiated by 10‐MV photon beam with dose rates of 100, 300, and 600 MU/min.

**FIGURE 3:**
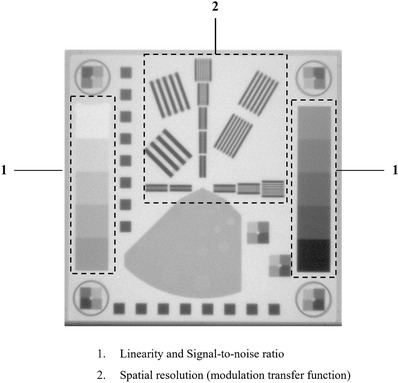
The image example of EPID QC phantom and the arrangement of physical evaluation items: (1) signal linearity and signal‐to‐noise ratio; (2) spatial resolution (modulation transfer function)

Another test was performed to compare the CR image quality used in clinical practice. The image equivalent to 70 MU, the dose of CR images currently used in clinical practice, was created with 12 images (300‐MU/min). The EPID QC phantom (Figure 3) consisted of absorbent copper. The image quality was evaluated by linearity of image signal values, signal‐to‐noise ratio (SNR), and low‐contrast resolution. The detailed construction and calculation algorithm in each image quality assessment of the EPID QC phantom were explained by Das et al.^20^

#### 2.8.1 Linearity

Copper wedges consist of five steps, covering the range of 0% to 50% absorption (0%, 5%, 10%, 15%, 20%, 25%, 30%, 35%, 40%, and 50%). The linearity curve is calculated from the mean gray values of 10 steps.

#### 2.8.2 Signal‐to‐noise ratio

The SNR is calculated from the average signal to the variance of the gray value in the copper step at each absorption level (*i* = 0%, 5%, 10%, 15%, 20%, 25%, 30%, 35%, 40%, and 50%). The calculation equation of the SNR is as follows:

$$SNR=\frac{\;Signal_{mean,i}}{\sqrt{\sigma_{i}^{2}}}$$

where the $Signal_{mean,i}$and the variance expressed as $\sigma_{i}^{2}$ are determined in each of the 10 gray level.

#### 2.8.3 Spatial resolution

The spatial resolution defined by modulation transfer functions (MTF) was determined using phantom line patterns. There are 14 blocks with 18 resolutions in a range of 0.167 to 3.5 linepair/mm. In this study, the MTF was determined by averaging horizonal and vertical directions of MTFs because the obtained results were almost similar. The MTF is calculated based on the image contrast between gray values of the absorber and gap in each of the 10 blocks.

### 2.9 Optimal thresholding in alert function

To detect intra‐fractional motion, an audible alarm sounds when the similarity metric calculated from the live and reference images exceed the arbitrary threshold. The similarity metric is determined by calculating the total absolute differences in each pixel that differ by ≥|±10%|between live and reference images. Determining the optimal thresholding value for detecting intra‐fractional motion, the LS was moved by 5, 10, and 20 mm to the anterior–posterior and superior–inferior directions and couch with the RANDO phantom lying on it was moved 5, 10, and 20 mm to the anterior–posterior direction. The optimal thresholding values to sound alert were obtained by manual adjustment of these values in each direction and displacement for LS and RANDO phantoms. For example, if the thresholding value is set below the optimal thresholding value Y to detect X cm movement, the alarm continues to sound, but if the threshold value is set above the optimal threshold value, the alarm does not sound.

### 2.10 Evaluation of scatter dose from imager

**FIGURE 4:**
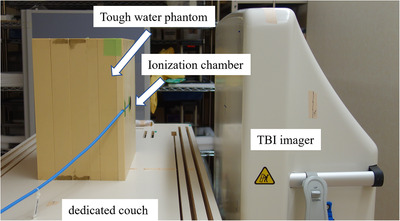
The arrangement for measuring the scattered radiation dose from the TBI imager. The parallel‐plate ionization chamber at a depth of 0 cm in a 40 cm thick tough water phantom placed on the dedicated couch. Its depth was changed to 0, 2, 5, and 20 cm, and measurements were performed with and without the TBI imager

The TBI imager is placed near the dedicated couch to include the entire lungs in the imaging area and to prevent the blurring effects caused by expanding the penumbra. To evaluate the scattered radiation dose due to prolonged irradiation, a 40‐cm‐thick tough water phantom (WD type, Kyoto Kagaku Co., Ltd., Kyoto, Japan) was placed on the dedicated couch so that its center was located at SAD of 400 cm (Figure 4). The surface of the parallel‐plate ionization chamber (Roos Chamber Type 34001, PTW‐Freiburg GmbH, Freiburg, Germany) was oriented toward the TBI imager and irradiated with 1000 MU of 10‐MV photon radiation at a dose rate of 300 MU/min. The depth of the parallel‐plate ionization chamber was changed to 0, 2, 5, and 20 cm, and measurements were performed with and without the TBI imager. The fraction of the irradiated dose and scattered radiation dose $\left(Dose_{SC}\right)$ was calculated using the following equation:

$$\begin{array}{ccc} & & Dose_{sc}\;\left(\%\right)\\ & & =\;\frac{Dose_{with\;imager}\left(nC\right)-Dose_{without\;imager}\left(nC\right)}{Dose_{without\;imager}\left(nC\right)}\times 100 \end{array}$$

where $Dose_{with\;TBI}$ is the measured dose with TBI imager and $Dose_{without\;TBI}$ is the without TBI imager. The polarizing voltage of the potentiometer (RAMTEC 1000PLUS, TOYO MEDIC CO., LTD., Tokyo, Japan) was set at 300 V.

### 2.11 Data processing

All the data were expressed as mean ± standard deviation. The image equivalent to 70 MU, the dose of CR images was created using MATLAB software (MathWorks, Natick, Massachusetts, USA).

## 3 RESULTS

### 3.1 Comparison the required time using CR to TBI imager

Table 1 shows the comparison of imaging procedures and required time for CR and TBI imager in TBI treatment with 12 Gy per six fractions. By repeating the procedures with carrying and reading the CR when LS positional accuracy is unacceptable increased the total time required by 6 min.

**TABLE 1:** Comparison of imaging procedures and required time for CR and TBI imager in TBI treatment with 12 Gy / 6 fraction (twice a day). Preparation of the thermoreversible immobilization device the day before treatment (Day 0) reduces the time required on the day of treatment using LS (Day 1 and 3). Asterisk (*) indicates required repeated procedures when LS positional accuracy is unacceptable; additional time is required for setup and readout CR when CR is used. DRR, digitally reconstructed radiograph; LS, lung shield; MV, megavoltage

		CR		TBI imager	
	Process	Procedure	Time	Procedure	Time
*Day 0*	*Preparation*	Patient positioning with the dedicated thermoreversible immobilization device	7 min	Patient positioning with the dedicated thermoreversible immobilization device	7 min
	*Imaging and verification of LS position*	* Bringing CRs into the room and setting CR on the dedicated couch	3 min	Setting TBI imager near the dedicated couch	2 min
		*Positioning the LS with reference to image on DRR (CR)	3 min	*Positionning the LS with reference to image on DRR (TBI imager)	3 min
		*Taking a MV image with CR	2 min	*Taking a MV image with TBI imager	2 min
		* Taking CR out of the room and readout CR image	3 min	*Quantitative evaluation of the position of the LS on MV image	2 min
		*Qualitative evaluation of the position of the LS on MV image	2 min		
	*Marking*	Marking the LS shape on the left and right side of the thermoreversible mmobilization device	15 min	Mark the LS shape on the left and right side of the thermoreversible mmobilization device	15 min
*Day 1 (3)*	*Preparation*	Patient positioning with the dedicated thermoreversible immobilization device	7 min	Patient positioning with the dedicated thermoreversible immobilization device	7 min
	*Imaging and verification of LS position*	* Bringing CRs into the room and setting CR on dedicated couch	3 min	Setting TBI imager near dedicated couch	2 min
		*Positioning the LS with reference to marking on the shell (CR image)	3 min	*Positioning the LS with reference to marking on the shell (image with TBI imager)	3 min
		*Taking a MV image with CR	2 min	*Taking a MV image with TBI imager	2 min
		* Taking CR out of the room and readout CR image	3 min	*Quantitative evaluation of the position of the LS on MV image	2 min
		*Qualitative evaluation of the position of the LS on MV image	2 min		
Total required time in each fraction with considering the repeated procedures based on the above asterisk (*) procedures. The maximum of repetition numbers was three.					
		Number of images taken		Number of images taken	
*Day 0*	*Total time*	1	35 min	1	31 min
		2	48 min	2	38 min
		3	61 min	3	45 min
*Day 1 (3)*	*Total time*	1	20 min	1	16 min
		2	33 min	2	23 min
		3	46 min	3	30 min

### 3.2 Accuracy of image scaling

In the vertical direction, the difference of the measured from the reference length for ‐2, ‐1, 0, 1, and 2 cm from the reference SAD was 4.7 ± 0.5, 4.5 ± 0.2, 3.6 ± 0.3, 4.0 ± 1.1, and 2.8 ± 0.5 mm, respectively. In the horizonal direction, the difference of the measured length was 3.6 ± 0.3, 3.1 ± 0.3, 2.2 ± 0.4, 2.2 ± 0.6, and 2.0 ± 0.4 mm, respectively. The difference error tended to decrease with increasing SAD.

### 3.3 Accuracy of measured lung shield displacement

**FIGURE 5:**
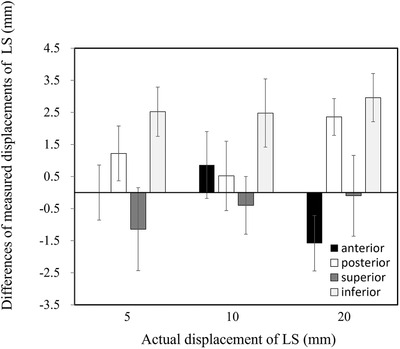
The difference of measured displacement for anterior, posterior, superior, and inferior directions from specified displacement (5, 10, and 20 mm) of the LS. LS, lung shield

Figure 5 shows the difference of the measured from specified LS displacement. Differences of the measured displacement for the anterior, posterior, superior, and inferior directions were 0.0 ± 0.9, 1.2 ± 0.9, ‐1.1 ± 1.3, and 2.5 ± 0.8 mm in a 5 mm displacement; 0.9 ± 1.0, 0.5 ± 1.1, ‐0.4 ± 0.9, and 2.5 ± 1.1 mm in a 10 mm displacement; and ‐1.6 ± 0.9, 2.4 ± 0.6, ‐0.1 ± 1.3, and 3.0 ± 0.8 mm in a 20‐mm displacement, respectively. The mean absolute displacement was 1.6 mm in all directions. The difference of the measured LS displacement was <4 mm in any displacements and directions, although a slightly large difference was observed in measured displacements in the inferior direction.

### 3.4 Image quality of TBI imager

**FIGURE 6:**
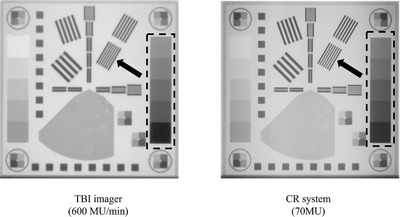
The example of EPID QC phantom image acquired with CR and TBI imager. Left side: 70‐MU equivalent image created by summing up 12 TBI imager images irradiated at a dose rate of 300 MU/min, right side: CR image irradiated at 70 MU. CR, computed radiography

Figure 6 shows an example of the EPID QC phantom image obtained using the TBI imager and CR system. In visual assessment, the image quality was not considerably different between the TBI imager and CR system. Images obtained from the TBI imager show slightly blurring in line patterns (black arrows) used for spatial resolution and reveal slight noise in the areas used for linearity and SNR (black dash rectangle).

#### 3.4.1 Linearity

**FIGURE 7:**
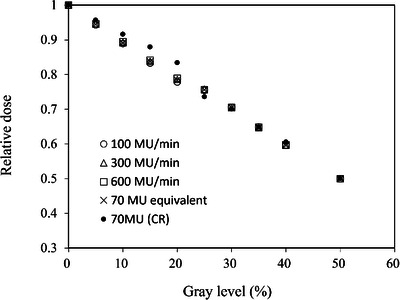
The linearity of changes in the gray level (%) with respect to the copper wedge absorption (%) in each dose level (100, 300, 600 MU/min, 70 MU equivalent, 70 MU with CR image)

Figure 7 shows linearity of changes in the relative dose based on the gray level (%) for all dose levels. No differences were observed among the dose rates with 100, 300, 600 MU/min, 70 MU (equivalent to CR image), and CR image.

#### 3.4.2 Signal‐to‐noise ratio

**FIGURE 8:**
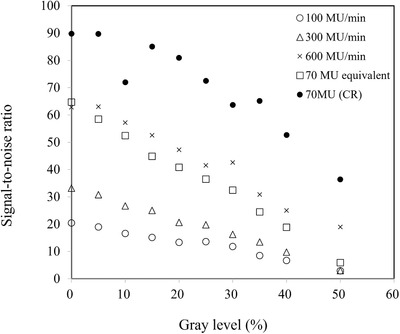
The change in the signal‐to‐noise ratio with respect to the gray level (%) in each dose level (100, 300, 600 MU/min, 70 MU equivalent, and 70 MU with CR image)

Figure 8 shows SNR changes based on the gray level (%) for all dose levels. SNRs were decreased with increasing gray level in all dose levels. A higher dose rate shows a higher SNR. The CR image shows superior SNR compared to images obtained using the TBI imager.

#### 3.4.3 Spatial resolution

**FIGURE 9:**
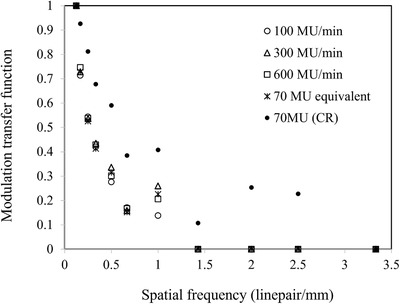
The modulation transfer function with respect to the spatial frequency (linepair/mm) of each dose level (100, 300, 600 MU/min, 70 MU equivalent, and 70 MU with CR image)

Figure 9 shows the MTF based on the frequency (linepair/mm) in each dose levels. No differences were observed among dose rates with 100, 300, 600 MU/min, and 70 MU equivalent. CR images had better MTF than images obtained using the TBI imager. Spike noises were observed in 1 linepair/mm for all dose levels in images obtained using the TBI imager.

### 3.5 Optimal thresholding in alert function

**FIGURE 10:**
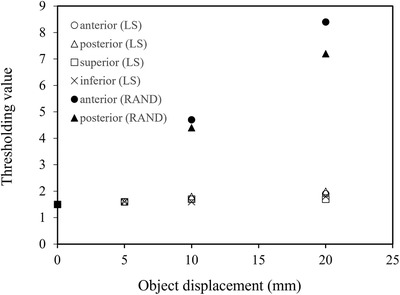
Thresholding values based on LS and RANDO phantom displacements. LS, lung shield. The average thresholding value to detect RANDO phantom displacement was 4.6

Figure 10 shows all the optimal thresholding values for the LS and RANDO phantom, which are dependent on the amount of displacement. In the LS, the optimal thresholding value to sound an alarm showed little changes in response to displacement changes. The RANDO phantom displacement has higher sensitivity than that of the LS. In RANDO phantom, the average thresholding value in anterior and posterior directions to detect 10 mm displacement was 4.6. Based on these results, the optimal thresholding value was finally determined to be approximately 5.0%, assuming a threshold value of 10 mm in body displacement. This value should be determined according to institutional criteria that consider acceptable intra‐fractional patient motion.

### 3.6 Evaluation of scattering radiation dose from the imager

**FIGURE 11:**
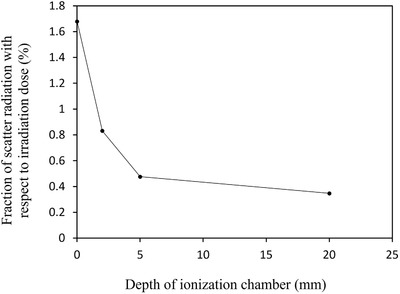
The increasing fraction of scattering radiation dose based on the depth of ionization chamber within tough water phantom due to the presence of TBI imager. The fraction of scatter radiation dose from the TBI imager decreased with increasing the depth of water‐equivalent phantom

Figure 11 shows the increasing fraction of scattering radiation doses due to the presence of the TBI imager. Maximum fraction of scattering radiation dose was 1.7% at the surface position and exponentially decreased as the depth increased.

## 4 DISCUSSION

The number of institutions maintaining a CR system tends to be decreasing because an EPID has been widely used to verify patient positioning instead of the CR system in general EBRT. Furthermore, the conventional CR system has the following disadvantages: time‐consuming and requires online monitoring of the patient during TBI. This is the first study that evaluated the setup accuracy, image quality, the optimal threshold value for alert function, and scattered radiation dose of the LS from imager as commissioning using a commercially available mobile TBI imager (CNERGY TBI). The total required time regarding the imaging procedure to position the LS with the CR and TBI imager depends on the number of repetitions, because of the additional procedures of carrying and reading the CRs when CR is used. The setup accuracy of the LS was within 4 mm while ensuring acceptable image quality through visual evaluation but was slightly inferior to the CR image. It would be possible to automatically detect unexpected patient motion error during prolonged TBI treatment by setting the optimal threshold value for the alert function. The estimated fraction of scattering radiation dose with the TBI imager increased by approximately 1.7%, which could not be considered to result in skin toxicity.

The use of EPID for TBI was firstly reported by Gladstone et al. ^21^ in 1993. Recently, Dipasquale et al. developed a completely movable and automatically EPID system consisting of amorphous silicon detectors for image‐guided TBI treatments.^19^ The CNERGY TBI is a commercially available stand‐alone mobile TBI imager consisting of CCD detectors.

The CR system has been used to position organ shield due to the absence of a dedicated EPID system accounting for long SSD treatment using a lateral beam, although it is a time‐consuming task. In the previous study investigated the total time requiring without image interpretation for CR and EPID image acquisition showed that with EPID was statistically significantly reduced compared to that with CR.^19^ The maximum requiring time in a repeated procedure without image interpretation were 5.1 min for CR and 2.5 min for EPID. These results were inconsistent with our study because there were several differences including the length of the flow line, the patient position (antero‐posterior field), and the fully automated EPID. However, the fact remains that use of EPID essentially reduces the time required for imaging.

To evaluate the setup accuracy of the LS, scaling accuracy was firstly verified using the scaling phantom with varying SADs. The residual error in calibration position (SAD, 358 cm) was 3.6 ± 0.3 mm and 2.2 ± 0.4 mm in the vertical and horizonal directions, respectively, and the difference in the measured distance decreased with increasing SAD. Then, the difference of the LS between the measured and intentional displacements was <4 mm in any displacements and directions. Gladstone et al. reported the accuracy in the LS positioning using the originally developed EPID system that median deviations in the right–left and superior–inferior directions were 0.0 cm (range, −3.3–2.0) and −0.3 cm (range, −5.5–3.5), respectively.^20^ The large variability occurred because the previous study measured actual patient images, whereas we used a RANDO phantom. Therefore, difference errors within a few millimeters would be acceptable, considering the actual patient positioning error. However, displacement differences in our results may be due to the inaccuracy of SAD measurement; thus, accurate SAD measurement at the calibration position would be necessary.

The previous study reported that image quality from CCD detector was superior to that from the CR system,^22^ while our results show that CR image quality was slightly superior to that of CCD. This difference may be caused by the low dose per image in CCD because the evaluation was made using the image cut from cine images. However, visual evaluation adequately identified lung regions and LS contour. TBI imager has a visually acceptable image quality with the same dose to CR images. Therefore, additional radiation exposure may be reduced if the number of images obtained using the CR system increased.

In the alert function, the threshold increased as the RANDO phantom displacement increased, and no difference in threshold values was observed depending on the direction of displacement of RANDO. Since the pixel values of the entire image are used to alert function, the thresholding value may not strongly　depend on the direction of the patient's displacement. However, patient size, lung size, and functionality may affect the threshold. Therefore, we determined the thresholding value to detect 1‐cm patient motion using a simple RANDO phantom to simulate actual treatment. Even if the threshold is not optimal due to differences in RANDO phantom and patient characteristics, calculated displacement based on manually drawn contour (e.g., lung and LS) can assist to detect intra‐fractional motion. Conversely, the displacement of a small object such as an LS could not be detected because the similarity metric calculated from an entire image area may have been less sensitive to small as the arbitrary regions of interest could not be set. To our knowledge, no reports have investigated temporary suspensions due to unexpected patient movement in prolonged TBI irradiation, although such suspensions often occur in routine clinical practice. Therefore, this system, which allows online monitoring of unexpected patient motion, would be useful and could contribute to a safe TBI treatment.

The maximum fraction of the scattered radiation to the irradiated dose was 1.68% at the patient surface when the TBI imager is positioned 50 cm downstream from the patient axis. The scattered dose at the surface was estimated to be 1.68 cGy on each patient side when irradiated with 2 Gy during the TBI treatment. Van Dam et al. reported that backscattered electrons can become clinically important (up to 20%) when the patient was positioned close to the wall of the treatment room.^23^ Our results were inconsistent with that of a previous study because of the difference in TBI imager composition and room wall. Therefore, our results could not cause any additional harmful effects by increasing the scattered radiation dose.

Finally, we developed the recommended quality assurance tests and its frequencies listed in Table 2 for ensuring the safe operation of the TBI imager in clinical practice. Most of them were referenced to the American Association of Physicists in Medicine Task Group 142 report and some literatures.^24^, ^25^

**TABLE 2:** A list of recommended quality assurance tests. Asterisks (*) indicate tolerance levels determined from AAPM TG 142.^24^ The tolerance levels of long‐term image stability and warm‐up characteristics in annual QA depend on types of imaging sensor. E2E, end‐to‐end

	Quality assurance tests	Tolerance
*Daily*	Operation check (alert, communication, visual inspection)	functional
	Image quality (visual evaluation of artifacts and image abnormality)	functional
*Weekly*	Scaling	≤2mm*
*Monthly*	Linearity	baseline*
	Contrast	baseline*
	Spatial resolution	baseline*
	Accuracy of in vivo dose calculation (if available)	within 10% ^27^
*Annual*	Long‐term image stability (or frequent in vivo dosimetry calibration is required)	decline of 0.04% day^‐1 [^ ^25^
	Verification of the threshold of the alert function (if available)	Baseline
	Confirmation of TBI imager distance	Baseline
	Confirmation of warm‐up characteristic	at least 40 min ^25^
	Indicator of lung block displacement through E2E test	≦5 mm (from our data)

The application of an EPID image include verification of the patient setup and assessment of the target and organ motion, treatment machine QA, and patient's in vivo dosimetry.^26^ During the TBI treatment using FIF and IMRT techniques, the field and MLC position should be confirmed based on the patient and collimator angle.^16^, ^17^, ^18^ Furthermore, dose uniformity in the TBI treatment is required to be within ± 10% in the whole body, and assessment using in vivo dose is recommended.^27^ However, CNERGY TBI used in this study is not capable of in vivo dose estimation. Our results showed excellent signal linearity, which may be expected to apply in vivo dosimetry during TBI treatment in a future study. This study has several limitations. First, accuracy assessment of LS positioning with TBI was not performed. Second, we did not compare setup times between CR systems and TBI imagers. Thus, they should be evaluated in a future study after clinical implementation.

## 5 CONCLUSION

This study performed commissioning of the commercially available mobile imager for TBI (CNERGY TBI) and presented the required validation items and baseline values. Results showed that the setup accuracy of the LS was within 4 mm in images with acceptable visual image quality to CR images. Automatic detection of unexpected errors would be possible; thus, safe treatment can be initiated in prolonged TBI treatments using the alert function.

## CONFLICT OF INTEREST

H. O has a research grant from Item Corporation.
